# Early adaptation to oxygen is key to the industrially important traits of *Lactococcus lactis* ssp. *cremoris* during milk fermentation

**DOI:** 10.1186/1471-2164-15-1054

**Published:** 2014-12-03

**Authors:** Marina Cretenet, Gwenaëlle Le Gall, Udo Wegmann, Sergine Even, Claire Shearman, Régis Stentz, Sophie Jeanson

**Affiliations:** Institute of Food Research, Norwich Research Park, Colney, Norwich, NR4 7UA UK; INRA, UMR1253, F-35000 Rennes, France; AGROCAMPUS OUEST, UMR1253, F-35000 Rennes, France; Research Unit Aliments Bioprocédés Toxicologie Environnements (UR ABTE) E.A. 4651, Université de Caen Basse-Normandie, Esplanade de la paix, 14032 CAEN cedex, France; 15 Albury Walk, Eaton, Norwich, NR4 6JE UK; 65 Rue de Saint-Brieuc, 35042 Rennes cedex, France

**Keywords:** *Lactococcus lactis*, Transcriptomics, Metabolomics, Stress response, Oxidative stress, Redox potential, Acidification, Growth

## Abstract

**Background:**

*Lactococcus lactis* is the most used species in the dairy industry. Its ability to adapt to technological stresses, such as oxidative stress encountered during stirring in the first stages of the cheese-making process, is a key factor to measure its technological performance. This study aimed to understand the response to oxidative stress of *Lactococcus lactis* subsp. *cremoris* MG1363 at the transcriptional and metabolic levels in relation to acidification kinetics and growth conditions, especially at an early stage of growth. For those purposes, conditions of hyper-oxygenation were initially fixed for the fermentation.

**Results:**

Kinetics of growth and acidification were not affected by the presence of oxygen, indicating a high resistance to oxygen of the *L. lactis* MG1363 strain. Its resistance was explained by an efficient consumption of oxygen within the first 4 hours of culture, leading to a drop of the redox potential. The efficient consumption of oxygen by the *L. lactis* MG1363 strain was supported by a coherent and early adaptation to oxygen after 1 hour of culture at both gene expression and metabolic levels. In oxygen metabolism, the over-expression of all the genes of the *nrd* (ribonucleotide reductases) operon or *fhu* (ferrichrome ABC transports) genes was particularly significant*.* In carbon metabolism, the presence of oxygen led to an early shift at the gene level in the pyruvate pathway towards the acetate/2,3-butanediol pathway confirmed by the kinetics of metabolite production. Finally, the MG1363 strain was no longer able to consume oxygen in the stationary growth phase, leading to a drastic loss of culturability as a consequence of cumulative stresses and the absence of gene adaptation at this stage.

**Conclusions:**

Combining metabolic and transcriptomic profiling, together with oxygen consumption kinetics, yielded new insights into the whole genome adaptation of *L. lactis* to initial oxidative stress. An early and transitional adaptation to oxidative stress was revealed for *L. lactis* subsp. *cremoris* MG1363 in the presence of initially high levels of oxygen. This enables the cells to maintain key traits that are of great importance for industry, such as rapid acidification and reduction of the redox potential of the growth media.

**Electronic supplementary material:**

The online version of this article (doi:10.1186/1471-2164-15-1054) contains supplementary material, which is available to authorized users.

## Background

Lactic acid bacteria (LAB) are historically defined as microaerophilic, Gram-positive organisms that produce lactic acid during homo- or heterofermentative metabolism of carbohydrates. Their ability to ferment hexoses makes them of great use in industrial applications to transform milk, meats and vegetables into fermented products. Their biosynthetic pathways are at the origin of a variety of flavors, textures and preservative qualities of fermented foods. Industrial applications of LAB rely on six key beneficial and nonpathogenic species [1]. Among them, *Lactococcus lactis* is the main source of mesophilic starter strains used for the production of different kinds of cheeses, fermented milks and cultured butter. The great economic importance of this species makes it a significant bacterial model for the study of anaerobic sugar catabolism [2, 3]. The formation of lactic acid from lactose, of diacetyl from citrate and its proteolytic activity are the three main lactococcal traits of interest for milk fermentation and aroma compound formation in dairy products, notably the underlying genes for these traits are all plasmid encoded.

*L. lactis* strains are divided in two subspecies designated as *L. lactis* subsp. *lactis* and *L. lactis* subsp. *cremoris*[4]. As an excellent model for research on bacterial metabolism and for the development of new applications, *L. lactis* genomics developed quickly with the availability of the first complete genome sequence of *Lactococcus* strain, *L. lactis lactis* IL1403 [5]. Currently, eleven complete *L. lactis* genomes are available with six strains belonging to the subspecies *lactis* and five to the subspecies *cremoris*. The complete genomic sequence of *L. lactis* subsp. *cremoris* MG1363 was released by Wegmann *et al.*[6] and revised by Linares *et al.*[7]. Most *L. lactis* mutants have been constructed from strain MG1363 and its derivatives. Many studies using these mutants focused on studying heme-respiration [8–10] or carbohydrate metabolism [11, 12] but few give global transcriptome profiles in response to environmental stresses. Recently, Carvalho *et al*. [13] presented the effect of acid stress on the glucose metabolism of non-growing cells of *L. lactis* MG1363 using nuclear magnetic resonance (NMR) and microarrays and De Jong *et al*. [14] performed temporal transcriptome analysis during growth of *L. lactis* MG1363 in milk. Global transcriptomic analysis in response to technological stresses, e.g. acidification and oxygen, have mostly been performed on strain IL1403 and its derivatives [15–19]. Specific studies of *L. lactis* MG1363 are still needed to be able to understand its responses to technological stresses.

Many physiochemical parameters, such as temperature, pH, water activity and pressure, affect *L. lactis* metabolism during cheese manufacture and are measured for quality control purposes. Additional parameters, such as the oxido-reduction potential, called redox potential (E_h_), and the concentration of dissolved oxygen [20], are of growing interest to the dairy industry. The concentration of dissolved oxygen is exponentially correlated to the redox potential [21] and changes constantly during cheese manufacturing. Oxygen is especially present at the very beginning of growth, when the milk is pumped and then stirred before coagulation. It has been shown that high initial levels of dissolved oxygen in milk can drastically delay acidification by *Lactococcus* starter strains [22, 23]. Cachon *et al*. [24] observed that *L. lactis* reduces milk prior to acidification. Reduction of milk by *L. lactis* is due both to oxygen consumption and a decrease of redox potential when oxygen disappears. Experimental data and genomic analyses indicate that *L. lactis* is able to cope with oxidative stress in a similar way to the aerobic model bacteria *Escherichia coli* and *Bacillus subtilis*[25–27]. *L. lactis* is relatively tolerant to oxygen and shows a great capacity to adapt to an oxygenated environment. In the presence of oxygen and heme, *L. lactis* is even able to respire and as a result, cell survival is improved and population is increased [8]. However, these heme conditions do not occur naturally. In an aerated environment, *L. lactis* is able to consume oxygen, resulting in an altered redox state and in a shifted carbon metabolism towards mixed fermentation [28]. However, studying the literature, several knowledge gaps can be identified, e.g. no study focused on the early exponential phase of growth when starter strains are exposed to oxidative stress, and no study took into account the redox potential values and the dissolved oxygen concentrations in relation to growth kinetics, metabolomic and transcriptomic data.

The aim of this study was to understand the adaptation of *L. lactis* to an initial oxidative stress, as encountered during the cheese-making process, at the transcriptional and metabolic levels in relation to the acidification kinetics and growth conditions. Whole genome transcriptome analysis of *L. lactis* MG1363 was performed at four time points (1, 5, 8 and 24 h of culture) during a 24 h period of growth and combined with a time-course quantitative analysis of produced metabolites by nuclear magnetic resonance (NMR) spectroscopy. A derivative of *L. lactis* MG1363, carrying the pLP712 plasmid, was grown in a medium containing lactose under two oxygen conditions: initial hyper-oxygenized medium (O2), to mimic the environment of a cheese-making process, and in oxygen-depleted medium (N2).

## Results

### *Lactococcus lactis*MG1363 underwent a drastic loss of culturability in condition O2

**Figure 1:**
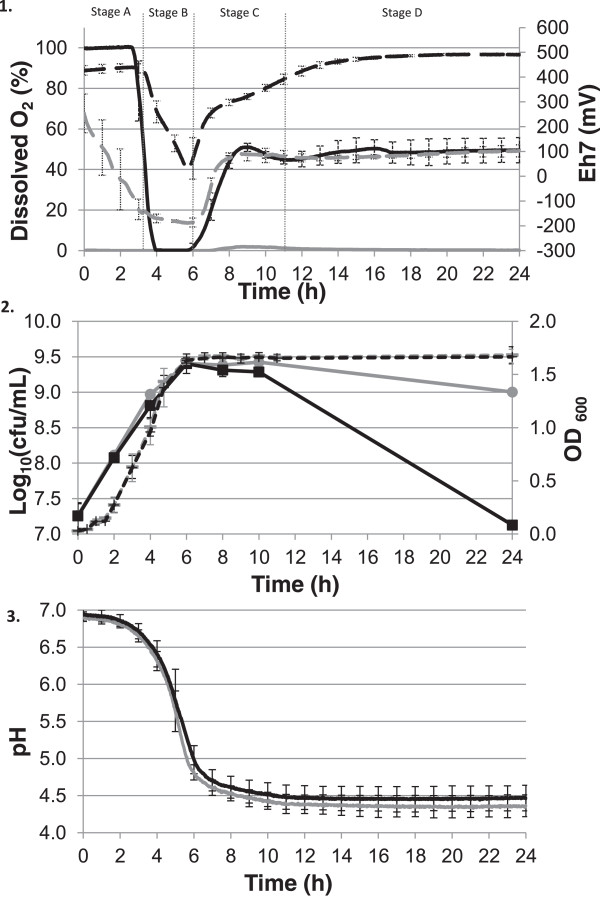
***Lactococcus lactis*** **ssp.** ***cremoris*** **strain MG1363 + pLP712 plasmid cultivated in LM17 at 30°C under O2 condition (black lines) and N2 condition (grey lines) for [1.] dissolved oxygen concentration (—) and redox evolution (−−−), for [2.] growth kinetics: enumerations (—) and OD**_**600**_ **(−−−) and for [3.] acidification kinetics.** Values are means of biological independent triplicates (n = 3) and bars represent the standard deviations.

The kinetics of growth (OD and enumerations), acidification (pH), reduction (E_h7_) and dissolved oxygen consumption (dissolved oxygen concentration) were monitored over 24 h for two conditions: initial hyper-oxygenized condition (O2) and no oxygen condition (N2) (Figure 1). Despite completely different gas conditions, no differences could be observed during the exponential growth phase, while substantial differences in culturability could be observed during the stationary growth phase. We defined 4 stages based on the oxygen concentration during growth under condition O2 (Figure 1). In stage A, the medium was initially saturated with oxygen (Figure 1.1) and the bacteria were in the early exponential growth phase (Figure 1.2), with minor changes in the pH (Figure 1.3) and the redox potential (Figure 1.1). In stage B, oxygen had totally been consumed and both culture media (under conditions O2 and N2) were anaerobic; bacteria were between mid- and late-exponential growth phase, the rate of acidification was at a maximum level and the redox potential decreased drastically since the oxygen concentration dropped abruptly to 0%. Until the strain MG1363 reached the stationary growth phase, its kinetics of growth and acidification was not affected by oxygen. Stage C started when the cells entered the early stationary growth phase reaching the maximum population density; acidification kinetics in conditions O2 and N2 were superimposed until a minimum pH was reached. In contrast to condition N2, the oxygen concentration in condition O2 increased again up to 50%, leading in turn to an increased redox potential. This increase of oxygen occurred because the bioreactor head-space was still filled with pure oxygen which dissolved into the LM17 medium and was presumably no longer consumed by the cells at this stage. Finally, during stage D the dissolved oxygen concentration stabilized at 50% of the initial value in condition O2 while there was still no oxygen detectable in condition N2. The *L. lactis* MG1363 was then in the late stationary growth phase, the minimum pH was reached (4.4 for condition N2 and 4.5 for condition O2) and the redox potential was stable (+100 mV for condition N2 and +490 mV for condition O2). The main difference between the two conditions occurred during this stage: the culturability loss was much higher under condition O2 (−2 Log_10_ CFU ml^−1^) than under condition N2 (−0.5 Log_10_ CFU ml^-1^).

### Predominance of differentially expressed genes at 1 h in the time-course analysis of *L. lactis*MG1363 transcriptome

**Figure 2:**
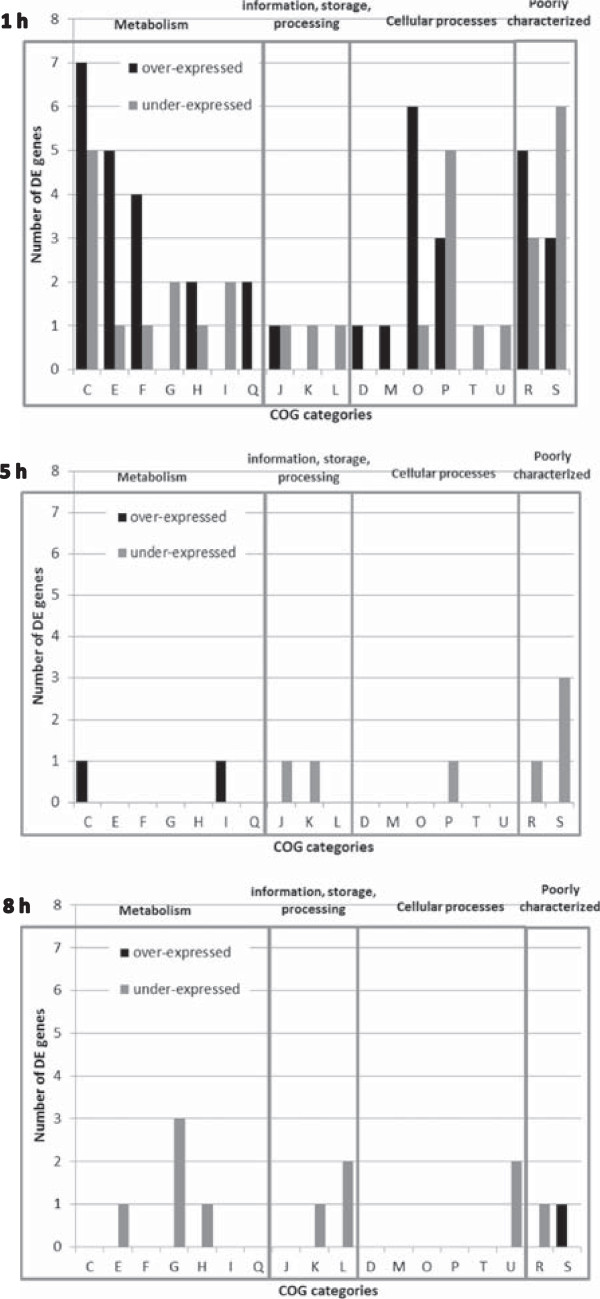
**Gene expression of strain MG1363: differentially expressed genes (over- and under-expressed), in O2 condition in comparison to N2 condition grouped by COG categories (letters) at 1 h, 5 h, 8 h of culture in LM17 at 30°C.** COG categories: Information storage and processing = > J “Translation, ribosomal structure and biogenesis”, K “Transcription”, L “DNA replication, recombination and repair”; Cellular processes = > D “Cell division and chromosome partitioning”, O “Posttranslational modification, protein turnover, chaperones”, M “Cell envelope biogenesis, outer membrane”, P “Inorganic ion transport and metabolism”, T “Signal transduction mechanisms”, U “Intracellular trafficking and secretion”; Metabolism = > C “Energy production and conversion”, G “Carbohydrate transport and metabolism”, E “Amino acid transport and metabolism”, F “Nucleotide transport and metabolism”, H “Coenzyme metabolism”, I “Lipid metabolism”, Q “Secondary metabolites biosynthesis, transport and catabolism”; Poorly characterized = > R “General function prediction only”, S “Function unknown”.

Transcriptomic analysis was performed on the whole genome of strain MG1363. Differentially expressed (DE) genes in condition O2 in comparison to condition N2 were determined at different time points corresponding to stage A (1 h), stage B (5 h), stage C (8 h) and stage D (24 h). All DE genes are described in the supplementary data (Additional file 1: Table S1) and DE genes discussed in the text are gathered in Table 1. Figure 2 represents the number of DE genes per known COG category, for each time point except 24 h (only one DE gene). The adaptation to oxygen strikingly occurred at 1 h (Figure 2.1) of culture (53 genes over-expressed and 37 genes under-expressed). All the COG categories were represented, indicating a global adaptation to oxidative stress. Moreover, COG categories C (energy production and conversion), E (amino acid transport and metabolism), F (nucleotide transport and metabolism) and O (post-transcriptional modification, protein turnover and chaperone) were specifically over-expressed in condition O2, clearly indicating that the cellular and energetic mechanisms were particularly affected at this early stage of growth. Only a small number of DE genes were observed at 5 h (Figure 2.2), most of which (11 out of 13) were under-expressed, probably because during stage B the absence of oxygen led to similar oxygen environment in both conditions O2 and N2, between 4 and 6 h of culture. In stage C (Figure 2.3), while dissolved oxygen increased again in the condition O2, only 23 genes were DE, of which 21 were under-expressed, indicating that the gene adaptation to oxygen observed in stage A did not occur despite the presence of oxygen.

**Table 1:** **Genes over- and under-expressed under oxygen conditions (condition O2) for the strain MG1363**

NomSpot	COG number	COG functional categories	***Gene name***	***Gene product***	1 h	5 h	8 h
llmg_1464	COG3527	Q	*aldC*	*alpha-acetolactate decarboxylase*	4.59		
llmg_1309	COG0028	EH	*als*	*acetolactate synthase large subunit*	3.64		
llmg_1700	COG4608	E	*choQ*	*choline ABC transporter ATP binding protein*	3.21		
llmg_1699	COG1732	M	*choS*	*choline ABC transporter permease and substrate binding protein*	3.61		
llmg_1737	COG0596	R	*cpo*	*non-heme chloride peroxidase*	2.27		
llmg_0346	COG1118	P	*fhuC*	*ferrichrome ABC transporter fhuC*	4.28		
llmg_0349	COG0614	P	*fhuD*	*ferrichrome ABC transporter substrate binding protein*	5.67		
llmg_0348	COG0609	P	*fhuG*	*ferrichrome ABC transporter permease protein*	4.15		
llmg_1702	COG1249	C	*gshR*	*glutathione reductase*	3.00		
llmg_0274			*llmg_0274*	*conserved hypothetical protein*	4.67		
llmg_0276	COG0656	R	*llmg_0276*	*oxidoreductase, aldo/keto reductase family*	3.52		
llmg_0281	COG1328	F	*llmg_0281*	*anaerobic ribonucleoside-triphosphate reductase*	6.11		
llmg_2143	COG1302	S	*llmg_2143*	*putative 20-kDa protein*	3.24		
llmg_2144			*llmg_2144*	*hypothetical protein predicted by Glimmer/Critica*	5.30		
llmg_2145			*llmg_2145*	*conserved hypothetical protein*	5.27		
llmg_0075	COG0095	H	*lplL*	*lipoate-protein ligase*	8.38		
llmg_1828	COG1169	HQ	*menF*	*menaquinone-specific isochorismate synthase*	2.69		
llmg_0951	COG0225	O	*msrA*	*peptide methionine sulfoxide reductase*	2.52		
llmg_1970	COG0822	C	*nifU*	*conserved hypothetical protein*	4.33		
llmg_0408	COG0446	R	*noxE*	*NADH oxidase*	22.93		
llmg_1543	COG0209	F	*nrdE*	*ribonucleoside-diphosphate reductase alpha chain*	16.70		
llmg_1544	COG0208	F	*nrdF*	*ribonucleoside-diphosphate reductase beta chain*	5.63		
llmg_0282	COG0602	O	*nrdG*	*anaerobic ribonucleoside-triphosphate reductase activating protein*	4.30		
llmg_1541	COG0695	O	*nrdH*	*Glutaredoxin-like protein nrdH.*	10.25		
llmg_1542	COG0716	C	*nrdI*	*Ribonucleotide reductase NrdI*	12.93		
llmg_0074	COG1071	C	*pdhA*	*pyruvate dehydrogenase E1 component alpha subunit*	5.96		
llmg_0073	COG0022	C	*pdhB*	*pyruvate dehydrogenase E1 component beta subunit*	5.03		
llmg_0072	COG0508	C	*pdhC*	*pyruvate dehydrogenase complex E2 component*	5.65		
llmg_0071	COG1249	C	*pdhD*	*pyruvate dehydrogenase complex E3 component*	2.84		
llmg_0629	COG1882	C	*pfl*	*formate acetyltransferase*		2.97	
llmg_0318	COG2077	O	*tpx*	*thiol peroxidase*	4.18		
llmg_0776	COG0492	O	*trxB2*	*thioredoxin reductase*	4.28		
**NomSpot**	**COG number**	**COG functional categories**	***Gene name***	***Gene product***	**1 h**	**5 h**	**8 h**
llmg_2432	COG1012	C	*adhE*	*alcohol-acetaldehyde dehydrogenase*	−7.45		
llmg_0100	COG2217	P	*cadA*	*cation-transporting ATPase*	−4.06		
llmg_1865	COG3104	E	*dtpT*	*di-/tripeptide transporter*	−2.11		
llmg_0758	COG0038	P	*eriC*	*Putative chloride channel protein.*	−2.04		
llmg_1551	COG2116	P	*fdhC*	*putative formate dehydrogenase*	−2.76		
llmg_1441	COG1053	C	*frdC*	*fumarate reductase flavoprotein subunit*	−4.24		
llmg_0993	COG0634	F	*hprT*	*hypoxanthine-guanine phosphoribosyltransferase*	−2.01		
llmg_1915	COG0247	C	*llmg_1915*	*putative Fe-S oxidoreductase*	−3.61		
llmg_0447	COG0674	C	*nifJ*	*Pyruvate:ferredoxin oxidoreductase and related 2-oxoacid:ferredoxin oxidoreductases, alpha subunit*	−11.19		
llmg_1514	COG2344	R	*rex*	*Redox-sensing transcriptional repressor rex.*	−2.73		
llmg_0640	COG1393	P	*trmA*	*Regulatory protein spx*	−2.15		
llmg_0429	COG0605	P	*sodA*	*superoxide dismutase*		−3.08	
llmg_1362			*orf44*	*hypothetical protein predicted by Glimmer/Critica*		−2.28	
llmg_1376	COG4227	L	*ltrC*	*LtrC*			−2.61
llmg_1377			*orf30*	*hypothetical protein predicted by Glimmer/Critica*			−2.09
llmg_1378			*orf29*	*hypothetical protein predicted by Glimmer/Critica*			−2.10
llmg_1379	COG2856	E	*orf28*	*Predicted Zn peptidase*			−2.06
llmg_1384			*orf24*	*hypothetical protein predicted by Glimmer/Critica*			−2.11
llmg_1387	COG5179	K	*orf21*	*hypothetical protein predicted by Glimmer/Critica*		−2.12	
llmg_1399			*orf10*	*hypothetical protein predicted by Glimmer/Critica*	−2.37		
llmg_1402			*orf8*	*hypothetical protein predicted by Glimmer/Critica*			−2.03

Fold-changes of selected differentially expressed genes of the strain MG1363 in O2 condition in comparison with N2 condition, from a whole-genome microarray analysis (n = 3). Genes were significantly (*p-value* < 0.5) differentially expressed with a FDR < 0.5 and a |fold-change| > 2. Top table is over-expressed genes; bottom table is under-expressed genes. Under-expressed genes; the ninth last genes are involved in the conjugation.

We will now focus on the main differences in gene expression between conditions O2 and N2, as determined by microarrays and further confirmed by quantitative real time PCR (RT-qPCR). The results are presented in Tables 1 and 2. Genes amplified by RT-qPCR were mainly selected as representatives of the key metabolic pathways activated or repressed in our experimental conditions in order to validate the global transcriptome analysis.

**Table 2:** **Gene expression confirmed using RT-qPCR under oxygen conditions (condition O2)**

					Ratio of gene expression O2/N2 as determined by:					
Locus tag	Gene	Gene product	Primer sequences		Transcriptome analysis			RT-qPCR		
			Forward	Reverse	1 h	5 h	8 h	1 h	5 h	8 h
llmg_0408	noxE	NADH oxidase	TTATGCCAAAGCAGAgGATTTT	GGAATAATTGGACGTGAACCTG	22.9	−3.2		26.7	2.0	
llmg_0074	pdhA	pyruvate dehydrogenase E1 component alpha subunit	AGGACGTATGGGATTCTTTGG	TTACCAAGTTGATGTCCACGAG	6.0	−2.3		7.7	2.8	4.1
**llmg_1541**	nrdH	Glutaredoxin-like protein nrdH.	AATTGTATGCAATGCAAAATGG	ATTACAGGAGCAGCTCGAAAAC	10.3		2.8	28.3	4.7	6.9
llmg_0776	**trxB2**	thioredoxin reductase	TGGTCTtTATGCGGCTTTTTAt	GGTAAAGATTTTGtGGcTGACC	4.3			13.3	2.4	
llmg_2432	adhE	alcohol-acetaldehyde dehydrogenase	GGTTCTGAAGTGACTCCATTTG	TCATAACAAACTCAGGGTCAACA	−7.5			−8.55		
llmg_1464	adlC	alpha-acetolactate decarboxylase	GGCTGACCAACCTTATTTTACA	TTCaGTgATGAAATTTTGGACA	4.6		3.7	11.8	2.3	4.8
llmg_1699	choS	choline ABC transporter permease	TTTTGCAAGTCACaGGAATTTTT	GGaAAAATCGCATAAACAACAAG	3.6			10.0	4.1	3.1
lmg2144	lmg2144	conserved hypothetical protein	TtGGcGGAATaaTAGGctgt	cGTgCgtTgaAgAtAgAGACC	5.3	−4.3		9.0		2.7
lmg0274	lmg0274	conserved hypothetical protein	CTCggTCATcGGAAAAGAAG	TTtGTaGCTTgcTGCCAGAG	4.7	2.3	2.4	5.3		
llmg_0100	cadA	cation-transporting ATPase	TTTGGCTTTAATCAGCCTTTTT	CCACCAGTAAAGTCGCAATAAT	−4.1				2.1	3.5
llmg_0349	fhuD	ferrichrome ABC transporter substrate binding protei	CATTAGGTGCAAATGTTGTTGG	TCAGGATTTTGAGCAATCAATTT	5.7			14.8	2.8	2.5
llmg_2050	tuf*	translation elongation factor EF-Tu	CACTCCATTCTTCGACAACTACC	AGGCATTACCATTTCAGTTCCTT						
llmg_0102	parA*	chromosome partitioning protein parA.	TTcTACAgCCgATtATtGTTCGT	TGGgATtGTTTCTAAtCCAGCTA						
llmg_0496	hllA*	HU-like DNA-binding protein	TTCAATTGATCGGTTTTGGTACT	TCAATGCTTTACCAGCTTTGAAT						

*tuf, *parA* and *hllA* expressions were used to normalised results.

Fold-changes of differentially expressed genes, in O2 condition in comparison with N2 condition, confirmed using RT-qPCR with a *p-value* < 0.05 and a |fold-change| > 2 (n = 3). Genes *parA*, *tuf* and *hllA* were internal control genes.

### The carbon metabolism is shifted to aerobic metabolism at an early stage of growth under condition O2

**Figure 3:**
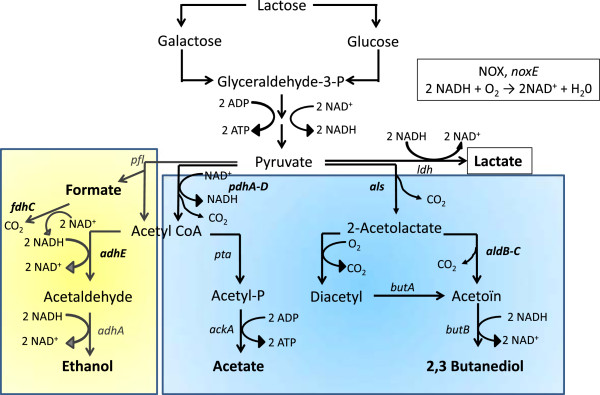
**The pyruvate metabolism of** ***Lactococcus lactis*** **.** *ldh:* gene coding for the lactate dehydrogenase; *pdhA-D:* genes coding for the pyruvate dehydrogenase complex; *pfl*: gene coding for pyruvate formate-lyase; *adhE:* gene coding for the acetaldehyde dehydrogenase; *adhA*: gene coding for the alcohol dehydrogenase; *pta*: gene coding for the phosphotransacetylase; *ackA*: gene coding for the acetate kinase, *als:* gene coding for catabolic and anabolic 2-acetolactate synthase; *aldB-C*: gene coding for the acetolactate decarboxylase; *butA:* gene coding for the diacetyl reductase; *butB:* gene coding for the acetoin reductase; *noxE*: gene coding for the NADH oxidase (NOX). Adapted from Oliveira *et al.*[29]. The blue area represents pathways up-regulated in O2 condition, and the yellow area represents pathways up-regulated in N2 condition.

As a type of homolactic bacteria, *L. lactis* produces mainly lactic acid from pyruvate, using glucose or lactose as carbon sources. However, other metabolites are simultaneously produced to a lesser extent from pyruvate using metabolic pathways dependent on culture conditions such as the oxygen level (Figure 3). *Lactococcus lactis* MG1363 displayed no differences in lactate dehydrogenase expression, under the two conditions O2 and N2. Besides this main pathway, the other pathways were affected under the condition O2. Genes *pdh*A-D, *als* and *aldC*, respectively encoding pyruvate dehydrogenase, acetolactate synthase, and alpha-acetolactate decarboxylase, were found to be over-expressed in condition O2 at 1 h (Table 1). Using RT-qPCR, genes *pdhA* and *aldC* were also found to be over-expressed at 5 h and 8 h (Table 2). It should be noted that the gene *lplL,* coding for a lipoate-protein ligase*,* which is adjacent to the *pdh* genes, also displayed an over-expression. On the contrary, under condition O2, genes *adhE* and *fdhC* (Table 1), respectively encoding alcohol acetaldehyde-dehydrogenase (AdhE) and formate dehydrogenase (FdhC), were under-expressed at 1 h. The gene *pfl*, encoding pyruvate formate lyase (PFL), responsible for the carbon flux from pyruvate to the formate/ethanol pathway (Figure 3), was over-expressed at 5 h (Table 1) indicating a switch from the aerobic to the anaerobic metabolism (Figure 3) during stage B. Finally, the gene *frdC,* coding for a flavoprotein involved in the reduction of fumarate to succinate, was under-expressed at 1 h.

**Figure 4:**
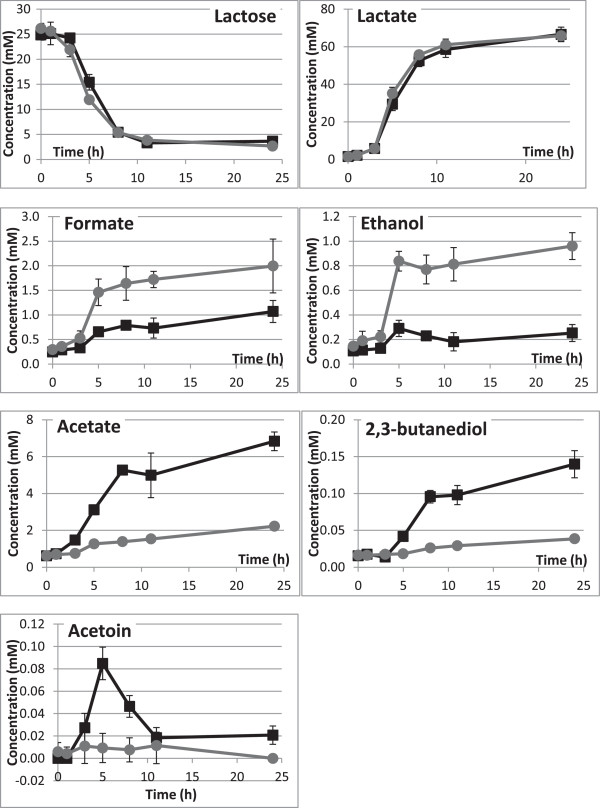
**Quantitative analysis by** ^**1**^ **H NMR of lactose and metabolites produced by** ***Lactococcus lactis*** **MG1363 in LM17 under O2 condition (■) and under N2 condition (**  **) over 24 hours of culture at 30°C.** Points are means of concentrations in mM (n = 3) and bars are standard deviations.

As a consequence of this early adaptation at the gene level, the metabolism was different under the two conditions 3 h into growth for most of the minor products derived from lactose. The metabolomics analysis performed using NMR analysis allowed to identify 27 metabolites (3 were only identified at the compound family level). Among them, 5 metabolites of major importance in the carbon metabolism of *Lactococcus lactis* had different kinetics of consumption or production under the two conditions. Figure 4 shows their kinetics (with the addition of lactose and lactate) at 7 time points over the 24 h-period. Lactose was not totally consumed at the end of the culture and its kinetics of consumption was not significantly different between the two conditions. Lactate was the main fermentation product in both conditions, with yields reaching 1.62 and 1.45 mol lactate per C_6−_mol of sugar consumed under conditions O2 and N2, respectively. On the contrary, minor products such as acetate, formate, ethanol and 2,3-butanediol displayed significantly different production kinetics under conditions O2 and N2 (Figure 4). While formate and ethanol were produced more under condition N2, acetate, 2,3-butanediol and acetoin were produced more under condition O2 (Figures 3 and 4). The differences in these kinetics were observable, at the 3 or 5 h time point, and increased steadily over the whole 24 h-period, except for acetoin, which was increasingly transformed into 2,3-butanediol from 5 h onwards.

### Genes involved in the response to oxidative stress were all DE early in the exponential phase, but not later

Genes involved in the response to oxidative stress and in electron transport were predominantly and highly expressed at 1 h (Table 1). These genes code for proteins involved in oxygen detoxification, electron transport and maintenance of the cell’s redox state. Among all, the gene which displayed the highest over-expression was *noxE* coding for the NADH oxidase responsible for the conversion of molecular O_2_ to H_2_O. Over-expression of *noxE* was confirmed by RT-qPCR at 1 h but not at 5 and 8 h (Table 2) indicating that this response only occurred in the early exponential phase. Genes involved in the transport of electrons displayed the same pattern: over-expression at 1 h but not later for gene *menF* involved in the synthesis of the menaquinone C (vitamin K2) and 3 out of 4 genes of the *fhu* operon coding for the ferrichrome ABC transporter (Table 1). However, high expression (14-fold) of the *fhuD* gene was confirmed by RT-qPCR at 1 h and to a lesser extent at 5 h and 8 h of culture (Table 2). The nucleotide reductases encoded by the *nrd* genes were part of the early adaptive response to hyper-oxygenized conditions, as all six *nrd* genes were over-expressed at 1 h with fold-changes ranging from 4- to 16-fold. RT-qPCR confirmed over-expression of the *nrdH* gene at 1 h, and - but to a lesser extent - at 5 and 8 h (Table 2). Other genes such as the genes involved in the control of the redox state of the cell, e.g. reduction of thiols (*trxB2, tpx, msrA*), reductases (*llmg_0276*) or a non-heme chloride peroxidase (*cpo*) which plays a role in the detoxification of hydrogen peroxide to water, were over-expressed at 1 h.

Conversely, oxygen-sensitive genes such as *nifJ* and *llmg_1915* were found to be under-expressed at 1 h. Transport proteins were also under-expressed: *cadA* coding for a cation transporting ATPase, *dtpT* and *eriC* coding for di-/tri-peptide transporter and for chloride transporter proteins, respectively. The under-expression of *cadA* was confirmed at 1 h using RT-qPCR, while later, at 5 h and 8 h, it was found to be over-expressed (Table 2). Genes encoding redox-sensing regulators such as *rex* and *trmA* were also found to be under-expressed under condition O2 (Table 1).

### Conjugation was repressed by the presence of dissolved oxygen both at the transcriptomic and phenotypic levels

Nine genes belonging to the *L. lactis* MG1363 sex-factor [6], a 60 kb chromosomally located conjugative element capable of high-frequency DNA transfer [30], were under-expressed in condition O2 (Table 1). The adaptive down-regulation of these genes by oxygen was most pronounced after 8 h of growth in agreement with the global adaptation at the transcriptomic level observed in this study. Interestingly, we observed that transfer frequency of the sex-factor was more than 5 times higher under static conditions (25 ± 2.0 × 10^−4^ transconjugants/donor) than under aerated conditions (4.6 ± 0.8 × 10^−4^ transconjugants/donor), thereby confirming that conjugal transfer is increased in limiting oxygen concentrations.

## Discussion

*Lactococcus lactis* is widely used as a starter in the dairy industry. During cheese-making, it is exposed to oxidative stress, e.g. during stirring, in the early stages of manufacture. *L. lactis* is known to be tolerant to oxygen. However, the non-respiratory response to oxygen, at the transcriptomic and metabolic levels, has never been described for the early stages of growth. The present results clearly showed that a high initial level of dissolved oxygen in LM17 does not affect the growth of strain MG1363 during the exponential phase, due to an efficient adaptive response at the transcriptional level occurring early in the exponential phase. On the contrary, when the level of dissolved oxygen increased again during the stationary growth phase, the cells suffered a dramatic loss of culturability, while the adaptive response at the transcriptional level was almost absent. This is the first study to simultaneously monitor growth and pH, but also oxygen concentration and redox values, which proved to be important as oxygen concentration varied a lot during growth.

### The growth and acidification of *L. lactis*MG1363 were not affected by oxygen during the exponential growth phase due to efficient oxygen consumption

The same growth and acidification kinetics were displayed under conditions O2 and N2 during the first hours, proving that strain *L. lactis* MG1363 has an efficient system to deal with oxygen. It has previously been shown that the presence of dissolved oxygen does not significantly affect growth of *L. lactis* strains either in LM17 [31], or in milk [23, 32]. In comparison to anaerobic conditions, the strain MG1363 could grow to an even higher population maximum (10% of biomass) under microaerobic conditions [33]. Acidification kinetics and final redox values were the same, so oxygen did not affect the main technological abilities of the strain MG1363. However, other properties could have been affected. In contrast, it has been reported that *L. lactis* subsp. *lactis* CHCC2862 acidified to a lesser extent under aerated conditions [31]. Furthermore, wild strains of lactococci can reach pH 5.5 in milk with 4–5 hours delay under the condition O2 in comparison to condition N2 [23]. The resistance to oxygen of the strain MG1363 is most probably due to its efficient oxygen consumption, clearing all oxygen from the media within 4 h. As a result, the redox potential showed a marked decrease. The minimum redox values (E_h7_) reached by strain MG1363 were typical for *Lactococcus* strains in milk with a minimum value between -200 mV and -280 mV [20, 23, 24, 32]. Interestingly, these values were also reached under condition N2, in the absence of dissolved oxygen, giving evidence of a reducing activity independent from the presence of oxygen as previously observed [32]. Reduction of the culture medium by *L. lactis* MG1363 indeed occurred in two consecutive steps under condition O2: an aerobic reduction with complete oxygen consumption during stage A and an anaerobic reduction with a decrease in E_h7_ values after oxygen has disappeared.

### *L. lactis*MG1363 displays multiple resistance systems in response to oxygen at 1 h

The most important and noticeable adaptation to oxygen was the early over-expression of detoxification and electron transport genes. All these genes code for enzymes protecting cells from damage provoked by reactive oxygen species. Evidence for two independent systems of reduction in *L. lactis* was shown [32]. The first one is NoxE; it is an oxygen-dependent system, responsible for the majority of oxygen consumption during the exponential phase, but its pH-sensitivity led to its inactivation during the stationary phase when pH decreased. The second system is oxygen-independent; it is composed of menaquinones (vitamin K2) that form part of the electron transport chain (MenC) and NoxAB, both responsible for the anaerobic reduction of the medium after oxygen consumption. However, in the present study, the gene *menF* (part of MenC system) was over-expressed at 1 h of culture at the same time as *noxE* and not later, suggesting that this enzymatic system is also part of the early adaptation to oxygen. These results are in accordance with Pedersen *et al*. [31], who found that *noxE* was the most DE gene in aerobic conditions, along with 5 of the *men* genes. In contrast to the work of Pedersen *et al*. [31], our results show that the *sodA* gene coding for the superoxide dismutase (SOD) was not DE at 1 h. The difference of experimental conditions may partly explain why the gene *sodA* was not DE in our conditions. In our conditions, the medium was initially saturated with dissolved oxygen while the medium was constantly aerated in Pedersen *et al.*[31]; the transcriptomic analysis was performed at OD = 1 in Pedersen *et al.*[31] instead of OD = 0.12 in our study. Furthermore, the strain used for the transcriptomic analysis in Pedersen *et al.*[31] was not strain MG1363. It has been recently shown that activities of NADH oxidase and SOD are strain dependent [34].

For the first time, the early over-expression of nucleotide reductase *nrd* genes of both classes (I and III) is described as a crucial part of the response to oxygen*.* The genes *nrdDEFGHI* were all over-expressed. The Nrd family of proteins is responsible for the reduction of ribonucleotide di- and tri-phosphates and thereby provides the building blocks for DNA replication and repair [35]. The NrdEFHI proteins are class I enzymes, which are oxygen-dependent, while the NrdDG system belongs to the class III enzymes, which can be active under anaerobiosis [36]. Under condition O2, the most over-expressed operons were *nrdHI* and *nrdEF* which is consistent with the fact that they are oxygen-dependent. The ribonucleotide reductase NrdEF uses NrdH as an electron transporter [35]. Surprisingly, the *nrdDG* operon, which encodes an anaerobic ribonucleotide reductase, was also over-expressed at 1 h, albeit to a lesser extent than the other *nrd* genes [36].

Another family of genes responsible for the efficient early adaptation to oxygen of strain MG1363 is the reductase and thioreductase family (*trxB2, llmg_0276, msrA, gshR*) maintaining the intracellular thiol balance [37]. In *Bacillus subtilis*, two genes, *trxA* (thioredoxin) and *trxB* (thioredoxin reductase), are transcriptionally induced under conditions of thiol-specific oxidative stress [38]. In *L. lactis*, these genes were not found to be over-expressed under aerated conditions at 4 h of culture by Pedersen *et al*. [31]. The *gshR* gene (glutathione reductase) was consistently over-expressed in O_2_-rich conditions as previously observed. Strain MG1363 is unable to synthetize glutathione but has been shown to display a high glutathione reductase activity on glutathione-rich medium such as LM17 medium [39], which could then protect against oxidative stress. The redox state is regulated by several genes when oxygen is present in the culture. The *rex* (redox-sensing transcriptional repressor) gene is under-expressed in our study. Rex, widely spread among bacteria [40], notably controls the expression of genes involved in energy metabolism and fermentative growth in several bacteria in response to the intracellular redox potential (NADH/NAD+ ratio). The *trmA* gene, under-expressed in our conditions, is one of the eight genes of the family of *trm* genes in MG1363 [6] and a member of the Spx family of proteins [41]. Spx of *B. subtilis* acts as a transcriptional regulator and senses redox conditions. It has been shown that a MG1363 *trmA* mutant was more resistant to stress with an increased proteolysis capacity [42]. Other genes involved in the electron transport by Fe(−S)-proteins such as *nifU* and *fhuCDG*, which code for ferrichrome ABC transporters for heme transport [9], were over-expressed. The *fhuCDG* genes had not been described as over-expressed under oxidative stress [43] or aerated conditions [31]. Rather our results suggest that these genes, as well as other genes involved in adaptation to oxygen such as *trxB2* (see above), are part of an early and transitory response of *L. lactis* to oxygen.

Finally, two genes, *choQ* and *choS,* coding for transport proteins of the osmoprotectant choline (osmotic stress response), were over-expressed suggesting overlap of stress responses as previously observed [44]. The over-expressed genes *llmg_2143, llmg_2144* and *llmg_2145* are similar to genes *ymgG, ymgH* and *ymgI*, respectively encoding two proteins similar to Gls24 family general stress protein and a transmembrane protein that were found to be induced by aerated conditions [31]. It is of interest that *llmg_2143* is located next to the integrase of a prophage in MG1363 and the gene environment may differ in other strains.

### Lactose fermentation of *L. lactis*MG1363 remains homolactic

Lactococci essentially produce lactic acid as a main end product [28]. In the present study, the MG1363 strain produced about the same concentration of lactate under the conditions O2 and N2. However, in agreement with the literature [28], part of the carbon flux or pyruvate flux is directed to mixed-acid fermentation pathway ending with metabolites such as acetate and aroma compounds (acetoin, diacetyl and 2,3-butanediol) under oxidative conditions. As PFL is irreversibly inactivated under aerated conditions [25], the pyruvate pool is deviated to the PDH (pyruvate dehydrogenase complex) and ALS (α-acetolactate synthetase) activities forming acetate and acetolactate, respectively. This pathway increases the ATP yield by acetate production. In parallel to the NADH oxidase activity, *L. lactis* oxidizes NADH into NAD^+^ with the two enzymes, α-acetolactate decarboxylase and 2.3-butanediol dehydrogenase, responsible for acetoin reduction into 2.3-butanediol [45]. The metabolic shift observed is likely a result of the under-expression of *frdC* and *adhE* and the over-expression of *pdhABD*, *als* and *aldC* genes*.* However, the production of these metabolites was observable two hours after the observed shift at the gene expression level. Interestingly, Pedersen *et al*. [31] did not mention the over-expression of these genes, suggesting this could be part of the very early adaptation of *L. lactis* to oxygen as mentioned above.

### *L. lactis*MG1363 adaption to oxygen disappeared in the stationary phase

In stages C and D, in condition O2 only, the oxygen concentration increased again to up to 50% of the initial value. Instead of a novel adaptation, we observed a drastic loss of culturability of the MG1363 cells. On one hand, such a loss of culturability does not exist in milk [23], not even for the MG1363 strain grown under the same conditions in milk (unpublished results). On the other hand, growth in M17 is most often monitored as OD and not colony forming unit enumerations [8, 12, 31, 37]. Consequently, to our knowledge, this drastic loss of culturability of *L. lactis* cells has not been reported before. Other strains of *L. lactis* (spp. *lactis* IL1403, spp. *cremoris* ML3 and SK11, both with a full plasmid complement) exhibited a loss of culturability after carbon exhaustion in M17 medium [46]. Strains MG1363 and ML3 (NCDO 763) are closely related and differ with regards to their chromosomal topography due to different chromosomal inversions [6]. In our study, carbon consumption stopped at very low concentrations (3 mM) but starvation was not observed during stage D under either condition. The loss of culturability observed in the present study may thus be due to oxidative stress only in combination with acid stress, and carbon source limitation might be a contributing factor. In milk, the loss of culturability does not occur because the increase of oxygen is extremely limited during the stationary growth phase and the lactose concentration was still high [23, 32].

### Surprisingly, conjugation is repressed in the presence of oxygen

The sex factor of the strain MG1363 contains 59 putative genes [6], 10 of which are predicted to encode proteins that are evolutionary conserved between 5 ancestrally related Gram-positive coccal conjugation systems [47] and are suggested to be part of a minimal set of genes required for conjugation. Among those genes, *orf24* and *orf28* were found to be under-expressed under conditions O2 at time point 8 h. We anticipated that in the presence of oxygen, a reduction of their expression and possibly of the other 7 genes would translate into a reduction of the sex-factor transfer efficiency. We show here that conjugal transfer is indeed increased 5-fold in limiting oxygen concentrations. These results are the first to describe the repression of the sex-factor conjugation under aerated conditions. This surprising result is in conflict with the common belief that conjugation facilitates adaptability of the cells to environmental stress [48].

## Conclusion

This study is the first aiming to investigate adaptation to oxygen by combining phenotypic (growth/acidification/conjugation), transcriptomic and metabolomic analyses over a 24 h-period of culture. It is very interesting to observe how uncorrelated the results of these different analyses could be. While no differences were visible at the phenotypic level during the exponential phase (same growth/acidification profiles independently of the aeration conditions), major differences occurred at the transcriptomic level, revealing a strong adaptation of *L. lactis* to the oxygen present. On the contrary, while transcriptomic profiles did not reveal major difference between conditions O2 and N2 in stationary phase, a strong loss of culturability was observed under oxygenated conditions. The present results were obtained for a lac^+^/prt^+^ strain directly derived from the strain MG1363. While strain MG1363 is regarded as a model organism representative of *Lactococcus lactis*, its behavior may not truly mirror that of an industrially used cheese starter culture. In all cheese-making processes, the milk is stirred during the first stages of the process, so oxygen is present early rather than later on. It is thus important to report that strains of the most used starter, *L. lactis*, are able to adapt to the presence of oxygen early in the exponential growth phase, when oxidative stress is most likely to occur. It is of major importance to understand how key technological abilities such as acidification and reduction of the medium are preserved in the presence of early oxidative stress.

## Methods

### Strains and culture conditions

The strain of *Lactococcus lactis* subsp. *cremoris* MG1363 [49] carrying the pLP712 plasmid (from the Institute of Food Research collection, Norwich, UK) was used for all fermentations. The plasmid pLP712 carries the protease gene and the *lac* operon [50] and allows the strain to growth on a medium containing lactose. A −80°C vial of the strain was cultivated in M17 liquid medium supplemented with 0.5% lactose and incubated at 30°C. After two successive cultures, the strain was grown overnight in 100 ml of the same broth medium. This overnight culture was used to inoculate the fermenters at 1% (v/v).

### Fermentation conditions

All fermentations were performed in one set of 2 l-bioreactors (LH Fermentation, series 2000) each with its own monitoring system. Fermentations were performed in filtered LM17 (M17 medium with 0.5% of lactose added). Before fermentation, the filtered LM17 was poured in the fermenters and gas conditions were set up before inoculation. For condition O2, pure O_2_ (Air Liquide) was sparged in the bioreactor until complete saturation with dissolved oxygen was reached. For condition N2, the bioreactor was sparged with pure N_2_ (Air Liquide) until no dissolved oxygen could be detected. Gas sparging was then stopped and inoculation was performed in both bioreactors. Fermentation protocols were carried out independently three times in LM17. The redox potential, the dissolved oxygen level and the pH were online monitored during 24 hours. All the sensors were autoclaved in the fermenters prior to fermentations. Dissolved oxygen concentration in the growth medium was measured using a polarographic electrode (Ingold). The pH and redox potential were monitored using autoclavable gel-filled electrodes (Mettler-Toledo). All electrodes were connected to an online meter (LH Fermentation, series 2000) that recorded the pH, dissolved oxygen and redox values every minute for 24 h. Growth was followed by measuring both the OD_600_ every hour until 11 h and at 24 h, and the cultivable counts every two hours until 10 h and 24 h, using a spiral platter on M17 + 1% lactose plates. Sampling for nuclear magnetic resonance (NMR) spectroscopy was performed at the same time than sampling for OD_600_ for growth and samples taken were stored at −80°C until NMR analysis.

The redox potential is the value measured from equilibrium of the redox couples in the medium. The measured value (E_m_) had to be corrected according to (i) the reference electrode and the temperature giving E_h_ (ii) the pH giving E_h7_. The electrodes have been treated and the calculation made as described in Abraham *et al*. [20].

### Quantitative analysis by nuclear magnetic resonance (^1^H NMR)

NMR was used to identify the presence, absence and concentration of several metabolites in growth medium. The supernatant samples were thawed at room temperature and prepared for ^1^H NMR spectroscopy by mixing 550 μl of spent medium with 200 μl phosphate buffer (0.2 M K_2_HPO_4_ and 0.038 M KH_2_PO_4_, pH 7.4) made up in 100% D_2_O and containing 0.06% sodium azide, and 1 mM TSP (sodium 3-(Trimethylsilyl)-propionate-d4) as a chemical shift reference. The sample was mixed and 500 μl transferred into a 5 mm NMR tube for spectral acquisition. ^1^H NMR spectra were recorded at 600 MHz on a Bruker Avance spectrometer (Bruker BioSpin GmbH) running TOPSPIN 2.0 software and fitted with a cryoprobe and a 60 slot auto-sampler. D_2_O was used as the internal lock. Each spectrum consisted of 64 scans of 32768 complex data points with a spectral width of 8389 Hz (14 ppm). The nuclear Overhauser effect spectroscopy (NOESY) pre-saturation experiment was used for each sample using a low power irradiation at the water frequency during the recycle delay to suppress the massive water signal. Spectra were Fourier transformed with 0.3 Hz line broadening, automatically phased and manually baseline corrected using the TOPSPIN software. The concentrations of metabolites were measured by a previously validated NMR targeted profiling method [51]. Spectra referenced to TSP (0 ppm) were uploaded in the Chenomx NMR suite version 6.0 software (Chenomx Inc.) and compounds identified and quantified by fitting library reference spectra of individual metabolites to the experimental spectrum.

### RNA and DNA extraction

Volumes of culture for RNA extraction were adapted to the concentration of cells (from 1.5 to 60 ml) in order to reach at least 10^9^ cells in total. Samples for RNA extraction were harvested directly in chilled phenol/ethanol (10%/90%) solution for a final concentration of 2% (v/v) of phenol in order to stop the transcriptomic activity. After incubation in ice-water for 30 min, the mixture was diluted 1:5 with a solution of citrate (1.8%) in water 98%/phenol 2%. All the samples were then centrifuged at 12,000 g for 15 min at 4°C, the cells were washed in 2 ml of the citrate water/phenol 2% solution, and centrifuged at 10,000 *g* for 20 min at room temperature. The cell pellets were then frozen at −80°C until RNA extraction.

Pellets were thawed at room temperature, and washed twice in 1 ml of TE buffer (10 mM Tris–HCl containing 1 mM EDTA•Na_2_, pH 8.0 (Sigma)). Total RNA was then extracted using the SV Total RNA isolation kits (Promega) according to the manufacturer’s protocol, except that cells were lysed using 100 μl/sample of a lysis buffer (1 ml of TE buffer +20 mg ml^−1^ of lysozyme +10 unit ml^−1^ of mutanolysin) and incubated at 37°C for 30 minutes. The RNA quantity was checked using 1 μl of the sample in a Nanodrop 1000 apparatus (Nanodrop) and RNA quality was checked using the RNA 6000 Nano kit on a Bioanalyser 2100 (Agilent Technologies). High-quality total RNAs (two sharp peaks for 16S and 23S ribosomal RNAs) were obtained for all the samples.

gDNA of *L. lactis* MG1363 was prepared from a 20 ml sample at an OD_600_ of 1 of a culture grown under static conditions on M17 supplemented with 1% lactose at 30°C. gDNA was extracted following instructions of Qiagen Genomic tip kit (Qiagen).

### Genomic expression analysis

Home-made microarrays containing 2459 PCR amplicon probes representing the coding sequences of the genome of *L. lactis* MG1363 were designed. Probes coding sequences from the lactococcal plasmid pLP712 were added [50]. The array design is available on the Arrayexpress database under the accession number A-MEXP-2216.

Ten μg of total RNA were labeled overnight at 42°C using 2 μl of Cy5-dCTP (Amersham Pharmacia), 4 μl of reverse transcriptase (AffinityScript, StrataGene) and 5 μg of random hexamers (Bioprime DNA labeling System, Invitrogen). Labeled cDNA was purified using QIA-Quick PCR purification kit (Quiagen) and eluted in 50 μl of RNAse-free water. For each microarray sample, 0.3 μg of gDNA were labeled with Cy3-dCTP using the same protocol. The expression profile of *L. lactis* MG1363 was determined by hybridizing Cy5-labeled cDNA corresponding to each sample of RNA with Cy3-labeled gDNA. Genomic DNA was used as a reference for normalization. At all the time points, microarray analysis was performed in triplicate (using independent biological samples).

For each time point, microarrays were hybridized overnight at 63°C with both labeled cDNA at a defined time point and labeled genomic DNA. Slides were then washed twice for 5 min in each of the following solutions: 2X SSC (where 1X SSC is 0.15 M NaCl/0.015 M sodium citrate) heated at 63°C, 1X SSC at room temperature and 0.2X SSC at room temperature. The slides were dried by centrifugation (1200 rpm, 5 min, room temperature) and scanned on a GenePix 4000A scanner (Axon Instruments). The fluorescent intensities for each cDNA/gDNA spot were measured using GenePix Pro 3.0 software (Axon Instruments).

### Statistical analysis

Median intensity of each spot was used as signal. To determine the expression profiles of *L. lactis* MG1363 under condition O2 and condition N2, data were analyzed with the R software [52]. Oligonucleotides with a very low signal for gDNA (corresponding to Signal Noise Ratio - SNR - lower than 2 in more than 75% of microarrays) were flagged and removed from further analysis. Data were first normalized per spot (cDNA signals divided by corresponding gDNA signals), Log_2_-transformed and then normalized per chip. Statistical analysis was performed using the ANOVA test considering a p-value and FDR lower than 0.05. Genes showing significant change in expression during fermentation with or without oxygen feed and a ≤0.5- or ≥2-fold change were considered as being differentially expressed.

Metabolomic data with growth/pH kinetics have been analyzed using a repeated series ANOVA (library nlme) in software R [52]. Models taking time and conditions (O2 and N2) as factors were tested. Time was introduced as a quantitative factor at degree-1 polynomial equation for the model. The results give the significance for each factor and their interaction (condition, time, time:condition). In this study, we focused on the significance of condition effect over the 24 h-time-course.

### Quantitative real time PCR (RT-qPCR)

In order to confirm transcriptomic analyses, quantitative RT-PCR experiments were carried out. Non-contamination of RNA sample by gDNA was confirmed by qPCR prior to cDNA synthesis. cDNA was synthesized using the high-capacity cDNA archive kit (Applied Biosystems) as recommended by the manufacturer. Quantitative real-time PCR was performed using an Opticon 2 real time PCR detector (BIORAD). The mixture contained power SYBR Green PCR master Mix (1X) (Applied Biosystems), each primer (0.5 μM) (sequences are in Table 2) and cDNA template. Thermal cycling consisted of 10 min at 95°C, followed by 40 cycles of 15 s at 95°C and 60 s at 60°C. A melting curve analysis (60°C to 99°C) was performed after the thermal profile to ensure specificity and PCR efficiency was calculated from the log-linear portion of the standard curves and comprise between 85% and 105% for every RT-qPCR run. Quantitative RT-PCR for all experimental time points was performed in triplicate (independent biological triplicates). Standard curves were generated to calculate the copy number of each gene in each sample. The most stable control genes were determined by geNorm among five potential genes*.* Real-time quantitative PCR data were normalized by geometric averaging of three internal control genes (*parA, tuf* and *hllA*). Gene expression was thus expressed as relative expression with regard to the normalization factor calculated by geNorm. Statistical analysis was performed using ANOVA test considering a p-value lower than 0.05 to identify genes showing significant change in expression with time.

### Conjugation as transconjugant frequency

To measure the sex factor transfer efficiency, the method described for plasmid transfer measurement in *Enterococcus faecalis*[53] and adapted by Stentz et al. [54] was used. Briefly, donor (strain FI8164) and recipient (strain FI9012) strains (from Institute of Food Research, Norwich, UK) were grown on M17 glucose 0.5% (v/v) at 30°C with tetracycline and rifampicin, respectively. Overnight cultures of both strains were diluted 100 times in fresh GM17 without antibiotic. After 4 h of growth, under both static (no agitation and limited head-space with 20 ml) and agitated (agitation 200 rpm and important head-space with 20 ml) for the donor strain and static conditions for the recipient, donor and recipient strains were mixed at a ratio 1:10. The mixture was vortexed for 30 s, centrifuged at 5000 g for 5 min in order to optimize donor-recipient mating, and left 1 hour at 30°C. Serial dilutions were made in PBS and plated on GM17 agar containing rifampicin (recipient and transconjugant selection), tetracycline (donor and transconjugant selection), and rifampicin and tetracycline (transconjugant selection). Transfer frequencies were calculated as transconjugants per donor.

## Electronic supplementary material

Additional file 1: Table S1: Fold-changes of differentially expressed genes of the strain MG1363, from a whole-genome microarray analysis, in O2 condition in comparison with N2 condition. Genes were significantly (*p-value*<0.5) differentially expressed with a FDR<0.5 and a |fold-change|>2. (XLSX 22 KB)
